# Intersubject variability and induced gamma in the visual cortex: DCM with empirical Bayes and neural fields

**DOI:** 10.1002/hbm.23331

**Published:** 2016-09-04

**Authors:** Dimitris A. Pinotsis, Gavin Perry, Vladimir Litvak, Krish D. Singh, Karl J. Friston

**Affiliations:** ^1^ The Picower Institute for Learning & Memory and Department of Brain and Cognitive Sciences Massachusetts Institute of Technology Cambridge Massachusetts; ^2^ The Wellcome Trust Centre for Neuroimaging, University College London Queen Square London WC1N 3BG; ^3^ Cardiff University Brain Research Imaging Centre (CUBRIC), School of Psychology, Cardiff University Park Place Cardiff Wales CF10 3AT United Kingdom

**Keywords:** empirical Bayes, random effects, dynamic causal modeling, neural fields, classification, Bayesian model reduction, gamma oscillations

## Abstract

This article describes the first application of a generic (empirical) Bayesian analysis of between‐subject effects in the dynamic causal modeling (DCM) of electrophysiological (MEG) data. It shows that (i) non‐invasive (MEG) data can be used to characterize subject‐specific differences in cortical microcircuitry and (ii) presents a validation of DCM with neural fields that exploits intersubject variability in gamma oscillations. We find that intersubject variability in visually induced gamma responses reflects changes in the excitation‐inhibition balance in a canonical cortical circuit. Crucially, this variability can be explained by subject‐specific differences in intrinsic connections to and from inhibitory interneurons that form a pyramidal‐interneuron gamma network. Our approach uses Bayesian model reduction to evaluate the evidence for (large sets of) nested models—and optimize the corresponding connectivity estimates at the within and between‐subject level. We also consider Bayesian cross‐validation to obtain predictive estimates for gamma‐response phenotypes, using a leave‐one‐out procedure. *Hum Brain Mapp 37:4597–4614, 2016*. © **The Authors Human Brain Mapping Published by Wiley Periodicals, Inc.**

## INTRODUCTION

The functional significance of cortical gamma band activity speaks to several important issues: including its ability to coordinate communication among neural populations, for example, those involved in attending to a particular stimulus [Fries, 2009], binding input features to cortical representations [Buzsáki and Chrobak, 1995; Gray et al., 1989] and mediating information transfer [Lachaux et al., 2005; Tallon‐Baudry et al., 1996]. In particular, gamma activity has on the one hand been used to characterize functional connectivity in cortical networks, for example, [Cabral et al., 2011] and on the other to study aberrant dynamics associated with potential pathophysiology. Gamma oscillations show a strong genetic dependency and may play an important role in disclosing aberrant neuronal processing in psychiatric diseases [Uhlhaas and Singer, 2012; van Pelt et al., 2012], such as autism [Dickinson et al., 2015] and schizophrenia [Gonzalez‐Burgos and Lewis, 2008]. They are thought to arise from a coordinated balance between cortical excitation and inhibition [Başar et al., 2015; Buzsáki and Wang, 2012] and are implicated a variety of cognitive processes like working memory [Miller and Wilson, 2008; Pesaran et al., 2002; Siegel et al., 2009] and visual attention [Womelsdorf et al., 2006].

Our focus here is on explaining intersubject variability in visual gamma responses in terms of cortical structure and function. In particular, we address the following question: which neurobiological mechanisms might explain differences in visually induced gamma oscillations recorded in different individuals and what is the role of intrinsic connections? This is the first application of empirical Bayes for dynamic causal modeling (DCM) [Friston et al., 2016] using real (electrophysiological) data. We call on neural field models that are particularly suited for modeling the spatial attributes of stimulus‐specific effects; such as the effect of stimulus size on spatially structured neuronal responses. Neural fields model the spatiotemporal convolution of afferent cortical input in a way that allows one to model spatial attributes of information processing; for example, the extent of horizontal (intrinsic) connections mediating the effects of surround suppression.

In this article, we consider the role of intrinsic or lateral interactions and gain control in mediating gamma responses to different visual stimuli. Our hypothesis is that individual differences in these parameters reflect variations in the excitation‐inhibition balance across individuals. Here, we use a parametric empirical Bayesian (PEB) model to obtain subject –specific estimates of these parameters after fitting group data. In particular, we exploit intersubject differences in gamma responses and use a hierarchical Bayesian model to describe both within and between subject effects. This entails the simultaneous optimization of model evidence (or variational Free Energy) across both levels. This allows us to identify the key components of cortical microcircuitry that determine gamma responses in a particular individual. We use a combination of biophysical models (neural fields) with DCM [Friston et al., 2003; Pinotsis et al., 2012]. This form of DCM offers a mechanistic understanding of brain function and individual differences in cortical responses. DCM has the potential to characterize pathophysiology that is expressed in terms of aberrant connectivity and synaptic plasticity [Dima et al., 2010, 2012; Roiser et al., 2013], or changes in consciousness level due to drug effects; e.g., [Boly et al., 2011, 2012; Moran et al., 2011; Muthukumaraswamy et al., 2013; Schmidt et al., 2013]. Here, we use DCM to ask which intrinsic connections determine intersubject differences in (stimulus‐induced) gamma responses by placing DCM in a hierarchical (empirical) Bayesian model of within and between‐subject effects. This hierarchical model also allowed us to establish the predictive validity of DCM with neural fields, by reversing the role of explanatory and response variables in a leave‐one‐out procedure. This cross‐validation procedure asks whether subject‐specific connectivity estimates can predict the frequency of observed gamma peaks.

This article comprises four sections. In the first, we provide a brief overview of DCM and the generative models used to explain electrophysiological data. We focus on neural field models and how they are used to predict spectral responses as measured by magnetoencephalography (MEG). This section includes a brief overview of hierarchical or empirical Bayesian modeling of within and between‐subject effects in DCM. The second section describes the visual paradigm, the cohort of participants, the experimental setup, MEG data acquisition and analysis. In the results section, we report a hierarchical PEB analysis to identify the intrinsic connections that best account for intersubject differences in gamma responses in the primary visual cortex. We then describe a Bayesian cross‐validation of the neural field DCM, using a leave‐one‐out procedure. The discussion reviews the relationship between stimulus size effects and gamma responses and considers the mechanisms that might underlie intersubject variability in induced gamma responses, in light of our empirical findings. In brief, our results endorse the notion that intrinsic connectivity—between excitatory and inhibitory pools of neurons within a cortical microcircuit—is a key factor in explaining individual differences.

## MATERIALS AND METHODS

### Modeling Induced Responses With DCM

DCM allows for a formal (Bayesian) characterization of local and global cortical interactions in terms of effective connectivity. These characterizations are based on the Bayesian model inversion and selection of neuronally plausible (biophysical) models. In the past years, several biophysical models have been implemented within the DCM framework, see [Moran et al., 2013; Pinotsis and Friston, 2014a]. Among these models, there exist cardinal distinctions; namely, the distinction between *convolution* and *conductance* models, the distinction between *neural mass* and *mean field* formulations and the distinction between *point sources* and *neural field* models. The first distinction pertains to the dynamics or equations of motion within a single population. Convolution models formulate synaptic dynamics in terms of a (linear) convolution operator, for example, [Moran et al., 2009; Pinotsis et al., 2012]; whereas conductance‐based models consider the (non‐linear) coupling between conductance and voltage, see e.g [Marreiros et al., 2010; Pinotsis et al., 2013]. The second distinction is between the behavior of a neuronal population or ensemble of neurons—as described with their mean or a *point probability mass* over state‐space. This contrasts with mean field approaches that model the ensemble density, where different ensemble densities are coupled through their expectations and covariances [Marreiros et al., 2015]. In other words, these models include a nonlinearity that follows from the interaction between first and second‐order moments [Marreiros et al., 2010; Pinotsis et al., 2013]. The final distinction is between models of populations as point sources (c.f., equivalent current dipoles) [Boly et al., 2011; David et al., 2006] and models that have an explicit spatial domain over (cortical) manifolds that call on neural fields [Pinotsis and Friston, 2014c]. These models are defined in terms of (integro) differential equations that describe cortical dynamics in terms of (spatially) distributed sources [Pinotsis and Friston, 2014b].

The current study uses neural field models that describe spatially distributed neural responses in terms of average (mean) depolarization, neglecting higher order moments. We used a neural field model for a single source defined by the equations

(1) $$\begin{array}{c} {\ddot{v}}_{1}+2\kappa_{1}{\dot{v}}_{1}+\kappa_{1}^{2}v_{1}=\kappa_{1}m_{e}(-d_{14}⊗\sigma (v_{4})+d_{11}⊗\sigma (v_{1})-d_{12}⊗\sigma (v_{2})+U)\\ {\ddot{v}}_{2}+2\kappa_{2}{\dot{v}}_{2}+\kappa_{2}^{2}v_{2}=\kappa_{2}m_{i}(d_{21}⊗\sigma (v_{1})+d_{22}⊗\sigma (v_{2})+d_{23}⊗\sigma (v_{3}))\\ {\ddot{v}}_{3}+2\kappa_{3}{\dot{v}}_{3}+\kappa_{3}^{2}v_{3}=\kappa_{3}m_{e}\left(-d_{32}⊗\sigma (v_{2})+d_{33}⊗\sigma (v_{3})\right)\\ {\ddot{v}}_{4}+2\kappa_{4}{\dot{v}}_{4}+\kappa_{4}^{2}v_{4}=\kappa_{4}m_{e}\left(d_{41}⊗\sigma (v_{1})+d_{44}⊗\sigma (v_{4})\right) \end{array}$$

**Figure 1:**
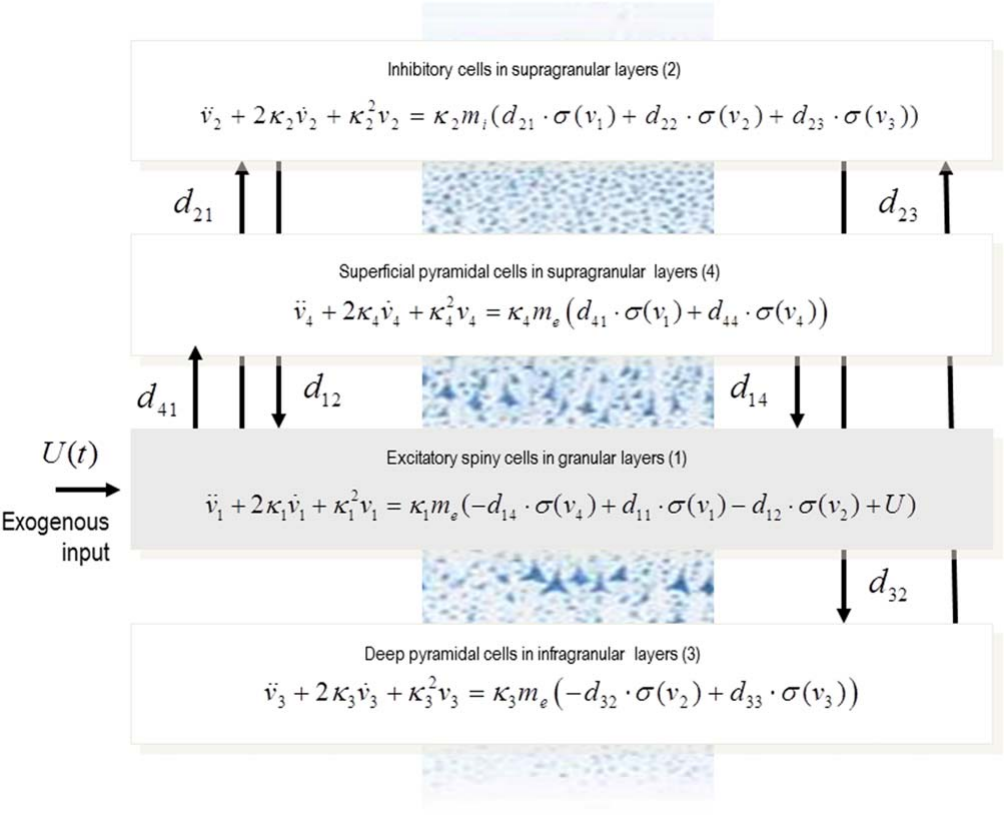
Schematic of the Canonical Microcircuit model with intrinsic connections. This schematic includes the differential equations describing the motion of hidden electrophysiological states. Each source is modeled with four populations constituting different cortical layers: these include two pyramidal cell populations that are generally presumed to be the sources of forward and backward connections in cortical hierarchies. Second‐order differential equations mediate a linear convolution of presynaptic activity to produce postsynaptic depolarization. This depolarization gives rise to firing rates within each sub‐population that provide inputs to other populations. [Color figure can be viewed at http://wileyonlinelibrary.com]

This model describes MEG responses that would be recorded from a local cortical patch in V1 with local extent of about 25 mm. Here, $v_{a}(t),a=1,\ldots 4$ denotes the expected depolarization in the *i*th population and $d_{ab}⊗\sigma (v_{b})$ is a spatiotemporal convolution describing the presynaptic input to the *i*th population from the *j*th. This convolution is defined by $(d_{ab}⊗\sigma (v_{b}))(x,t)=\int \int d_{ab}(x-x^{\prime },t-t^{\prime })\sigma \circ v_{b}(x^{\prime },t^{\prime })dx^{\prime }dt^{\prime }$ where the sigmoid function is given by $\sigma (v_{b})=\frac{1}{1+\exp(r(\eta -v_{b}))}$ and *r* is the synaptic gain and η is the postsynaptic potential at which half of the maximum firing rate is achieved. This is a function of postsynaptic depolarization in the *b*th population that is multiplied by intrinsic connection strengths $d_{ab}$ between the two populations. Here, $m_{i}$ and $m_{e}$ are synaptic parameters controlling the maximum postsynaptic responses (for the inhibitory and excitatory populations respectively) and $\kappa_{a}$ are the corresponding rate‐constants of postsynaptic filtering (c.f., decay). See Figure 1 for a schematic of this model. Below, we use [Eq. (1)] above to obtain cross spectral densities after linearizing around a fixed point, see [Pinotsis et al., 2014] for more details.

### Spectral Responses of Neural Field Models

Local field potential or MEG recordings often show oscillatory responses that reflect summed activity from excitatory and inhibitory pools of neurons under various input stimuli and in several cortical regions [Hauck et al., 2007; Katzner et al., 2009; Xing et al., 2009]. Our focus is on visually induced responses and individual differences in cortical oscillations in the gamma band. The spectral responses of neural field models can be summarized in terms of transfer functions for a range of physiological parameters. These transfer functions can be obtained in a straightforward manner from the dynamical equations in Eq. (1)—and can be regarded as a representation of cortical dynamics in the Fourier domain. In short, the transfer functions depend on the spatial and synaptic parameters of a neural field model that determine the spectral properties of observed activity (like peak frequency and power).

Neural field DCMs are based on likelihood models that map from hidden (neurobiological) parameters to measured responses that are sampled with a single or multiple sensors—like MEG or ECoG data [Pinotsis et al., 2012]. Here, we model (beamformed) MEG data with a four‐layer canonical microcircuit (CMC) field model of a single source depicted in Figure 1.

The CMC is a laminar‐specific intra‐cortical architecture that describes how information flows within and between cortical layers. This model is based on findings in primary visual cortex [Douglas and Martin, 1991] but recent work, for example, [Lefort et al., 2009] indicates that similar microcircuits exist in other regions—such as somatosensory and motor cortex (for a fuller discussion, see [Bastos et al., 2012; Pinotsis et al., 2014]). This circuit comprises four layers or populations: excitatory spiny stellate (SS) input cells (1), inhibitory interneurons (II) (2), deep excitatory pyramidal cells (3), and superficial excitatory pyramidal cells (4). The model also includes biophysical parameters like intrinsic interlaminar and horizontal connectivity. These parameters are summarized in Table 1 in terms of their prior means, which will be used for single subject inference and studying group effects in subsequent sections.

**Table 1:** Model parameters

Parameter	Physiological interpretation	Prior mean
$\kappa_{1},\kappa_{2},\kappa_{3},\kappa_{4}$	Postsynaptic rate constants	1/2, 1/35, 1/35, 1/2 (ms^−1^)^a^
$\alpha_{11},\alpha_{14},\alpha_{12}$ $\alpha_{22},\alpha_{21},\alpha_{23},\alpha_{33}$ $\alpha_{41},\alpha_{32},\alpha_{44}$	Amplitude of intrinsic connectivity kernels (× 10^3^)	108, 45, 1.8 9, 162, 18, 45 (a.u) 36, 18, 9
$c_{ab}$	Spatial decay of connectivity kernels	$\{\begin{array}{cc} 0.6 & a\neq b\\ 2 & a=b \end{array}$ (mm^−1^)^b^
r,η	Parameters of the postsynaptic firing rate function	0.54, 0 (mV)^2^
*S*	Conduction speed	0.3 m/s^3^
ϕ $q_{1},q_{2},q_{3},q_{4}$	Dispersion of the lead field Neuronal contribution weights	$\sqrt{2}/16$ (mm) 0.2, 0, 0.2, 0.6^c^
$a_{u},a_{n}$	Exogenous white input, channel‐specific white noise (log –scale)	0, 0
$\beta_{u},\beta_{n}$	Exogenous pink input, channel‐specific pink input (log –scale)	0, 0

a Wendling et al., 2000.

b Kandel et al., 2000.

c The values for *q* are just prior assumptions about the relative (percentage) contributions from populations of SS (1), II (2), Deep Pyramidal cells (3), and SP cells (4). We assume that major contributions to the observed signal come from the pyramidal cell populations, see Pinotsis et al., [2014] for more details.

The spatial parameters assume the cortical patch has a diameter of ℓ=25 mm.

These parameters determine the behavior of a cortical source, whose activity is modeled as a (filtered) response to (endogenous) neuronal fluctuations. The spectral profile of this response generally has one or more spectral peaks, including a peak in the gamma region (when the parameters are set to their prior means). The origin of this gamma activity is an active area of research and could be due to random membrane depolarizations of individual units due to noisy inputs [Brunel and Wang, 2003; Burns et al., 2011]. In our framework, this random activity is described by the spectral density of endogenous neuronal fluctuations $g_{u}(k,\omega )=U(k,\omega )U{\left(k,\omega \right)}^{*}$ that produce observed (cross spectral) responses at sensors *l* and *m* according to the following likelihood model:

(2) glmi(ω)=Γi(θ(1))+gn(ω)+ɛ(1)Γi(θ(1))=∑kTl(k,ω)gu(k,ω)Tm(k,ω)†Tr(k,ω)=Lr(k,φ)Q⋅T(k,ω,θ(1))gn(ω)=αn+βn/ωgu(ω)=αu+βu/ωRe(ɛ(1))∼N(0,Σ(ω,λ))  Im⁡(ɛ(1))∼N(0,Σ(ω,λ))

Here, $L_{r}(k,\varphi )$ is the Fourier transform of the lead field of the *q*th sensor,^1^ † denotes the conjugate transpose matrix and $Q=[q_{1},q_{2},q_{3},q_{4}]$ is a vector of coefficients that weights the contributions of each neuronal population to the observed MEG signal. Here, $g_{u}(\omega )$ is a spatiotemporal representation of fluctuations or inputs driving induced responses, which we assume to be spatially white and a mixture of white and pink temporal components. These contributions are based on differences in anatomical properties and the lead field configuration of each population (e.g., inhibitory neurons do not generate a large dipole), where each electrode or sensor has its own sensitivity profile, reflecting the topographic structure of the underlying cortical source.

Equation (2) describes the predicted cross spectra as a function of the power of underlying neuronal fluctuations $g_{u}(\omega )$ and transfer functions $T(k,\omega ,\theta^{\left(1\right)})$ that depend on model parameters at the first or within‐subject level: $\theta^{\left(1\right)}$. The transfer functions $T(k,\omega ,\theta^{\left(1\right)})$ are the Fourier transform of the impulse response or first‐order Volterra kernel associated with (ordinary or integro) differential equations in Eq. (1) and are given explicitly in the Appendix. These transfer functions describe how each of the four populations responds to neuronal fluctuations, where the model parameters describe the connectivity architecture mediating responses, the observation function $\varphi \subset \theta^{\left(1\right)}$ and the spectra of the inputs and channel noise, $\{\alpha_{n},\alpha_{u},\beta_{n},\beta_{u}\}\subset \theta^{\left(1\right)}$. In this (single source ‐ single sensor) setup, Eq. (2) models gamma rhythms as the bandpass‐filtered output of the cortical circuit depicted in Figure 1, whose input is a mixture of white and pink noise; compare [Burns et al., 2011].

In summary, Eq. (2) expresses the data features g(ω) as a mixture of predictions and sampling errors ${ɛ}^{\left(1\right)}$ with covariance Σ(ω,λ). Gaussian assumptions about these sampling errors provide the likelihood model at the first (within‐subject) level: $p(g(\omega )|\theta^{\left(1\right)})$. The predictions are themselves a mixture of predicted cross spectra $\overset{⌢}{g}(\omega )$ and channel noise $g_{n}(\omega )$. This concludes the description of the likelihood model. We next consider how this likelihood model is placed within a hierarchical model of responses from multiple subjects

### Hierarchical or Empirical Bayesian Modeling

In a Bayesian context, hierarchical models are known as *empirical Bayesian models*. Common examples are PEB models, in which random effects are assumed to be Gaussian (as above). Effectively, hierarchical models equip subject‐specific or first‐level models with empirical priors that optimally shrink parameter estimates to a group mean. This approach distinguishes between (first‐level) models that generate subject‐specific data and (second‐level) linear models that contain explanatory variables at the between‐subject level.

Equation (2) defines a model of predicted cross spectral densities that maps from hidden (neurobiological) parameters to observed MEG responses at the first (within‐subject) level. To explain intersubject variability, this model is supplemented with a mapping from group means to subject‐specific estimates. This defines an empirical Bayesian model that can be optimized efficiently using Bayesian model reduction [Friston and Penny, 2011]. Bayesian model reduction entails the estimation of a posterior density over hidden model parameters for a reduced model (defined in terms of a prior density) using just the posterior density estimated from a full model (with a complete set of parameters). This approach is based on the following general form of hierarchical model and implicit (empirical) priors:

(3) $$\begin{array}{c} \ln p(g(\omega ),\theta^{\left(1\right)},\theta^{\left(2\right)})=\sum_{i}\ln p(g{\left(\omega \right)}_{i}|\theta^{\left(1\right)})+\ln p(\theta^{\left(1\right)}|\theta^{\left(2\right)})+\ln p(\theta^{\left(2\right)})\\ p\left(g{\left(\omega \right)}_{i}|\theta^{\left(1\right)}\right)=N(\Gamma_{i}(\theta^{\left(1\right)}),\Sigma_{i}(\theta^{\left(1\right)}))\\ p\left(\theta^{\left(1\right)}|\theta^{\left(2\right)}\right)=N(\Gamma (\theta^{\left(2\right)}),\Sigma (\theta^{\left(2\right)}))\\ p\left(\theta^{\left(2\right)}\right)=N(\eta ,\Sigma ) \end{array}$$

In our case, the first (subject‐specific) level [Eq. (3)] is given by [Eqs. ((2)−2.6)] and intersubject variability [Eqs. (3.2−3.3)] is modeled by

(4) $$\begin{array}{c} \theta^{\left(1\right)}=(X⊗I)\theta^{\left(2\right)}+{ɛ}^{\left(2\right)}\\ \theta^{\left(2\right)}=\eta +{ɛ}^{\left(3\right)} \end{array}$$

Here, *X* is a design matrix describing between‐subject effects and ${ɛ}^{\left(2\right)},{ɛ}^{\left(3\right)}$ represent random effects at higher levels (i.e., intersubject variability and uncertainty about the group mean). To model differences among subjects, we have to specify phenotypic differences among subjects (e.g., age or clinical diagnosis). Here, we will characterize subjects in terms of their characteristic gamma responses using three proxies (see below). These explanatory variables or phenotypes constitute the design matrix *X*. The Kronecker tensor product with the identity matrix X⊗I means that we have a second level parameter for every second level (phenotypic) variable and every first level (connectivity) parameter. This means one can identify the combination of connectivity parameters and phenotypic variables that best explains intersubject variability. This is addressed using Bayesian model reduction (or comparison) over (second level) models (after inverting a DCM for each subject). We perform an exhaustive search over all possible (reduced) models where one or more second level parameters have been removed. In other words, we create a large model space that includes all reduced models; in which every combination of effects is suppressed, using precise shrinkage priors.

## EXPERIMENTAL DESIGN AND PROCEDURES

The data modeled below were taken from a previous study (Experiment 1 of [Perry et al., 2013]) in which MEG was used to measure the gamma response to visual gratings of three different sizes (2°, 4°, and 8°) in 12 healthy volunteers (3 female, 9 male; mean age: 30.67 years, range: 20–43 years.). A detailed description of the methods can be found in [Perry et al., 2013] and is briefly summarized here.

### Experimental Procedure

Stimuli were stationary, vertically oriented black/white square‐wave gratings, with a spatial frequency of 3 c.p.d; presented at maximum contrast and masked by a square window that varied in size by condition. During each trial, a red square (∼0.2° in width) was present continuously and participants were instructed to maintain fixation on the square throughout. Stimuli were positioned so that their top right‐hand corner always coincided with fixation. This ensured that the stimuli were presented in the lower‐left visual quadrant, thereby precluding source cancellation across primary visual cortex representing different visual quadrants.

Each trial comprised a 1,500 ms baseline period, in which only the fixation square was present. This was followed by a stimulus for a random duration between 1,000 and 1,500 ms, followed by a 1,000 ms response period; resulting in a total trial time of 3,500–4,000 ms. Gratings were presented at one of three different sizes—2°, 4°, and 8°—and participants were instructed to indicate which of the three different sizes had been presented by pressing one of three buttons using their right hand during the response period (i.e., after grating offset). Participants viewed 100 trials per condition (300 trials in total), and trials were presented in random order.

### Data Acquisition

Whole‐head MEG recordings were acquired using a 275‐channel CTF radial gradiometer system sampled at 1,200 Hz. An additional 29 reference channels were recorded for noise cancellation purposes, and the primary sensors were analyzed as synthetic third‐order gradiometers [Vrba and Robinson, 2001]. For source localization, a multiple local‐spheres forward model [Huang et al., 1999] was derived by fitting spheres to the brain surface extracted by FSL's Brain Extraction Tool [Smith, 2002]. Estimates of the three‐dimensional distribution of source power were derived for the whole head at 4 mm isotropic voxel resolution for each participant. Manual inspection of the SAM images demonstrated that each participant had a single positive peak of activity in the right occipital cortex. A time series of the response at the location of the peak in each trial was generated by spatially filtering the sensor‐level data through the corresponding beamformer weights at that location. These “virtual sensor” time series were then modeled with DCM. For more details on data pre‐processing, we refer the reader to [Perry et al., 2013].

### Size Effects and Gamma Responses

Several studies have concluded that gamma responses depend crucially on various input features, for example, orientation [Frien et al., 2000], the presence of luminance contours [Swettenham et al., 2013], motion [Swettenham et al., 2009], speed [Friedman‐Hill et al., 2000], and contrast [Henrie and Shapley, 2005; Perry et al., 2015]. In particular, the spatial structure of the visual stimulus has been shown to affect gamma band activity [Bauer et al., 1995; Gieselmann and Thiele, 2008; Lima et al., 2010]. In our earlier work [Perry et al., 2013], we tested the relationship between the gamma‐band response and the size of visual grating stimuli in humans using MEG. We found that the absolute magnitude of the gamma‐band response increased with size, despite considerably varying across participants. This stimulus‐dependence, could have important implications for the role of gamma oscillations in neuronal processing [Ray and Maunsell, 2010] and extra‐classical receptive field effects have been shown to depend on stimulus context and spatial configuration, for example, [Akasaki et al., 2002; Mizobe et al., 2001]. Surround suppression is a (well‐known) reduction of neural responses that emerges as the size of the stimulus becomes larger and encroaches on regions outside the classical receptive field [Allman et al., 1985; DeAngelis et al., 1994]. This phenomenon is implicated in a variety of perceptual tasks, where the spatial structure of the visual input is important, including figure‐background segmentation [Supèr et al., 2010], contour integration [Hess et al., 2013] or depth perception [Kim et al., 2015].

**Figure 2:**
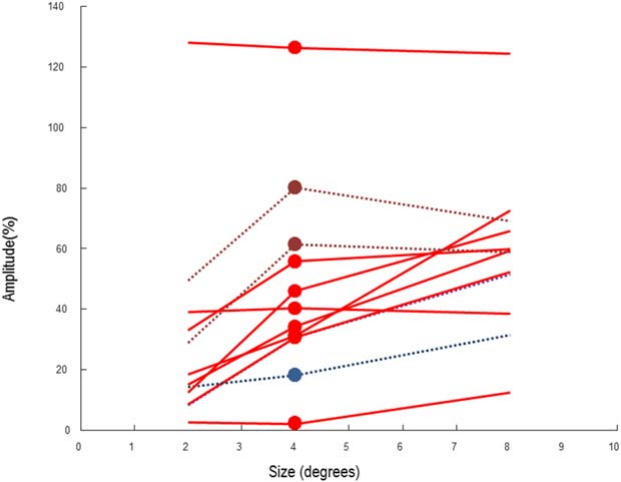
Graph showing the amplitude of the gamma‐band response as a function of stimulus size (depicted in linear scale). Dashed curves show exemplar subjects with the following two sorts of responses: subjects with the largest response for the smallest stimuli had a saturating response (magenta) while those with a weaker response showed an increasing response up to the largest stimulus size (blue). Responses are presented as percentage changes relative to baseline (%). [Color figure can be viewed at http://wileyonlinelibrary.com]

This article focuses on individual differences in gamma responses elicited by vertically oriented gratings of different sizes. Here, we use visually induced V1 responses in the 30–80 Hz range reported in [Perry et al., 2013]. These authors showed that sustained activity varied across individuals while varying stimulus size. Perry et al. first tested whether the frequency of this gamma‐band response varies with stimulus size. A repeated measures ANOVA indicated that, contrary to expectation, there was no significant effect of size on frequency (*F*(2,22) = 0.034, *P* = 0.72). Next the authors asked whether the amplitude of the gamma‐band response was affected by the size of the stimulus. To test this, they identified the peak frequency of response independent of condition (i.e., from the mean amplitude spectra across all trials) and measured the change in source amplitude (relative to baseline) at this frequency for each condition (only data from 0.5 to 1 s were included). Figure 2 shows the results for different individuals. The difference in gamma response to stimulus size was found to be significant using a repeated‐measures ANOVA (*F*(2,22) = 18.1, *P* = 0.00002), where, quantitatively, average gamma‐band response rose approximately linearly with log stimulus size.

Figure 2 shows the effects of stimulus size on gamma responses for different subjects. These responses either show an approximately linear (monotonic) increase in the gamma‐band response or a saturating response with increasing size, akin to surround suppression. In other words, participants showed different local response functions in the gamma band that might be due to individual differences in the local connectivity architecture within visual cortex. Below, we use three data features (phenotypes) to address the following question: which neurobiological mechanisms could explain this intersubject variability? Motivated by the above analyses of Perry et al. and similar studies in the cognitive neuroscience literature, we focussed on peak frequency (across all trials), peak amplitude (the largest amplitude in any of three conditions), and the change in amplitude with stimulus size (the ratio between the largest amplitude from either the 4° or 8° conditions and the amplitude from the 2° condition). Note that the results in Figure 2 were based on responses to stimuli of different sizes, which are used to characterize intersubject differences. However, in our DCM analyses, we only modeled responses to the same stimuli (i.e., 2°). This means that the results in Figure 4 below pertain to a single stimulus size. Our question in this article was, therefore, whether between‐subject differences in responses to stimuli of varying sizes could explain intersubject differences in intrinsic connectivity induced by the same stimulus size.

In summary, Figure 2 shows that subjects with the largest response to the smallest stimuli had a saturating response while those with a weaker response showed an increasing response up to the largest stimulus size [Perry et al., 2013]. This suggests systematic intersubject differences in gamma responses that may be due to differences in local (intrinsic) connectivity. We hypothesized that these intersubject differences in gamma responses could be explained by differences in the excitation‐inhibition balance that mediate surround suppression (see discussion). These mechanisms are, in turn, mediated by changes in intrinsic connectivity and inter‐ and intra‐laminar coupling within visual cortex.

In what follows, we tested this hypothesis, with a special focus on identifying the particular connections and neuronal populations responsible for intersubject differences. We inverted the hierarchical model in Eq. (3) and use Bayesian model reduction to identify the intrinsic connections that account for intersubject differences in gamma responses in V1. This enabled us to test the prediction that connections involving II (in a pyramidal‐interneuron gamma or PING network) are the key determinants of intersubject variability. In total, our (first level) model comprises four populations and 10 connections. At the second level, this means there are 10 parameters for each phenotypic or between subject explanatory variable. These parameters are given in Table 2. This table describes all the intrinsic connections in the neural field model between the four populations which are depicted as vertical arrows in Figure 1: SS, Superficial Pyramidal (SP) cells, II, and Deep Principal (DP) cells. Parameters 1,4,7 and 10 describe recurrent self‐excitation (gain) of all the populations and parameters 3–6 and 9 describe connections involving II that are known to play an important role in the generation of gamma oscillations [Traub et al., 1997].

**Table 2:** Correspondence between the parameters of Figure 6 and the CMC parameters of Figure 1 (SS = Spiny stellate, SP = Superficial pyramidal, II = Inhibitory interneurons, DP = Deep pyramidal)

**Parameter number**	**1**	**2**	**3**	**4**	**5**	**6**	**7**	**8**	**9**	**10**
CMC parameters (Fig. 1)	$d_{11}$ SS→SS	$d_{14}$ SP→SS	$d_{12}$ II→SS	$d_{22}$ II→II	$d_{21}$ SS→II	$d_{23}$ DP→II	$d_{33}$ DP→DP	$d_{41}$ SS→SP	$d_{32}$ II→DP	$d_{44}$ SP→SP

This table lists the parameters in Figure 6 in terms of the connections in the cortical microcircuit in Figure 1 (depicted with black vertical arrows).

## RESULTS

Our goal was to identify which (connectivity) parameters—in particular, intrinsic connections—subtend intersubject variability in observed gamma responses. To make inferences about intersubject differences, it is necessary to use a hierarchical model that accommodates within and between subject effects, such as Eq. (3). In what follows, we used routines that have been developed recently for empirical Bayesian models [Friston et al., 2016]; in particular DCM for group studies, to ask whether intersubject differences in gamma responses could be explained by, or explain, intersubject differences in intrinsic connectivity.

Our hierarchical model treated intrinsic connectivity as a random (between‐subject) effect, which was modeled by adding random Gaussian effects to subject‐specific parameters as is standard in DCM models for Cross Spectral Densities [Moran et al., 2009; Pinotsis et al., 2012]. The intrinsic connectivity and other fixed effects (i.e., the remaining DCM parameters) then generate observed spectral density responses for each subject as described above. Crucially, the inversion of this hierarchical model allows one to test different hypotheses at the between‐subject level. In this setting, all model parameters (in Table 1 above) are fitted for each subject, including the time constants of GABAergic channels that have been shown to play a prominent role in generating gamma oscillations [Brunel and Wang, 2003].

**Figure 3:**
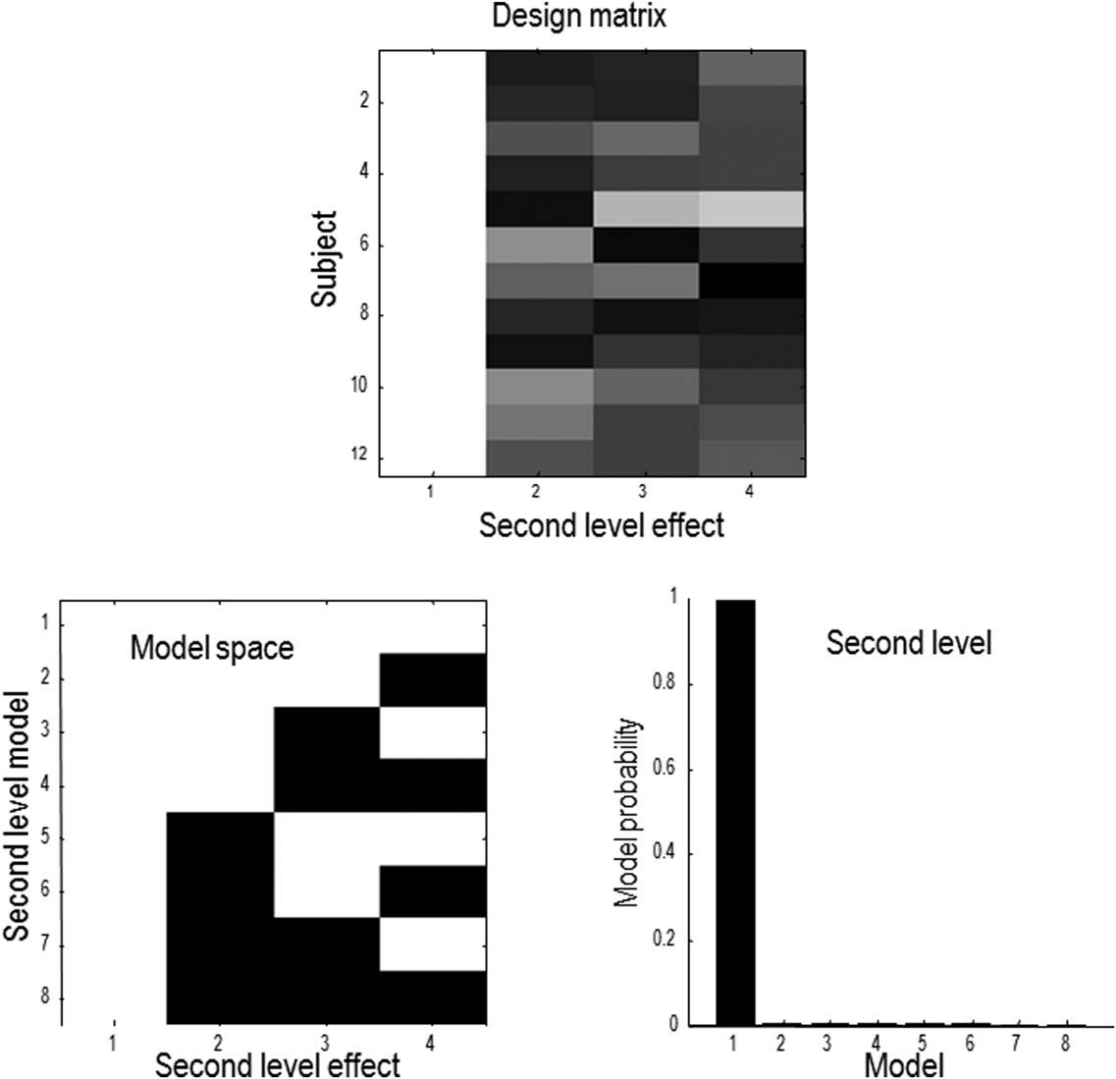
Above: Design matrix containing the explanatory variables or between subject (random) effects; these include a constant term and three parametric variables based on each subject's responses. Left: model space. This represents the model space we considered. This space contains all combinations of second level effects encoded by the design matrix. Right: the resulting posterior probability over models shows that the first model with all four effects (constant term and three between subject effects) had the greatest posterior probability. In the lower panels, black corresponds to one and white is zero (an arbitrary grayscale is used to show the second level design matrix).

The synaptic or connectivity parameters describing cortical microcircuitry are considered to mediate systematic group differences (cf., Figure 2). To identify the specific parameters that are responsible for observed variability in gamma responses, we consider all possible hypotheses; each hypothesis or model is scored by its evidence. In other words, we compare (second level) models, in which between‐subject effects may or may not be expressed in different combinations of intrinsic connections. This model comparison can be performed very efficiently using Bayesian model reduction as described elsewhere (see [Friston et al., 2016] for details). The models or hypotheses are specified in terms of the (second level) parameters of a design matrix of (between‐subject) explanatory variables; in exactly the same way as one would specify between‐subject effects in an analysis of covariance [see Eq. (3) and Fig. 3].

### Model Specification and Inversion

**Figure 4:**
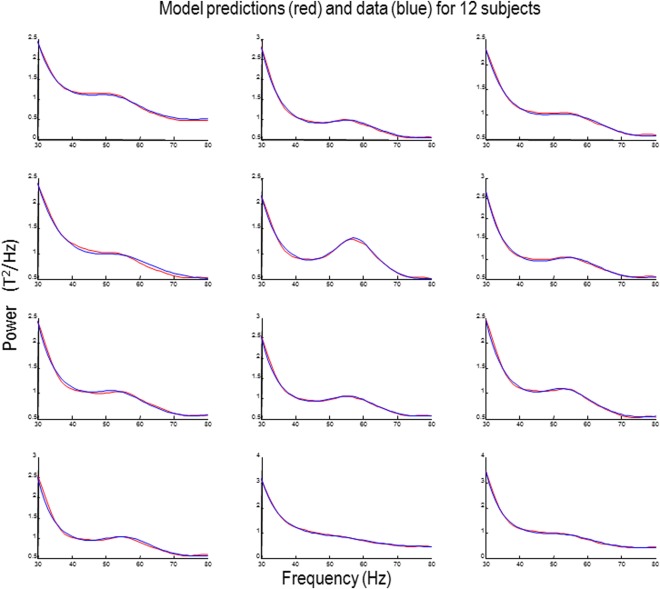
Observed and predicted spectral responses obtained following model inversion for 12 subjects included in this study. These plots show the spectral responses (cross spectral densities) obtained during the 2° stimulus condition. The agreement between the model predictions (red line) and empirical spectra (blue line) is self‐evident. [Color figure can be viewed at http://wileyonlinelibrary.com]

To specify the hierarchical model, we need to decide which phenomenological variables (phenotypes) we will use to characterize subject‐specific differences. In this application, we used three proxies to describe phenotypic variations between subjects; namely, the change in amplitude of gamma responses with increasing stimulus size, the peak frequency over all stimuli and the amplitude of gamma responses (based on the maximum amplitude across stimuli): These are the proxies used in [Perry et al., 2013] and serve as phenotypes that allow one to characterize intersubject variability in visually induced gamma responses. These are the candidate explanatory variables (occupying the columns of the design matrix in the top of Fig. 3) that might account for between‐subject (second level) variability. At the first (within subject) level, we used the single source model with full connectivity, compare, Figure 1. Exemplar empirical responses (blue lines) and model fits (red lines) are shown in Figure 4.

To ensure that one or more of the between‐subject explanatory variables were necessary to explain observed responses, we first compared all combinations of the three explanatory variables and assessed the model evidence, pooled over subjects (using **spm_dcm_bmc_peb.m**).^2^ This implicitly optimized the model space over the first and second levels. This means that one or more explanatory variables could explain individual variability, resulting in 2^3^ = 8 plausible hypotheses or models: see bottom left of Figure 3, after inverting a DCM for each subject (using **spm_dcm_peb_fit.m**). Crucially, this empirical Bayesian approach is implemented so that one can compare different second level models without having to repeat the inversion of each subject's DCM. This can be thought of as a generalization of the standard summary statistic approach.

Figure 3 shows the results of this analysis. The top panel shows the second level design matrix of explanatory variables (phenotypic proxies). In the lower panels, black corresponds to one and white is zero (an arbitrary grayscale is used to show the second level design matrix). The first column is a group mean or constant term, while the subsequent three columns correspond to change, frequency and amplitude of global gamma responses. The ensuing (second level) models are shown schematically on the lower left. These models represent all combinations of the three explanatory variables. In the lower panels, black corresponds to one and white is zero (an arbitrary grayscale is used to show the second level design matrix). In other words, we evaluate the evidence for models with and without each of the thirty second level parameters. Recall that there is one parameter for each of the 10 intrinsic connections of the neural field model (see Table 2 and vertical arrows in Figure 1) and each of the three phenotypes. This means that there is a very large number of models in the model space. This ensures a fairly exhaustive and efficient model optimization, see Friston et al. [2016] for details. The posterior probability over these models (based on the variational free energy approximation to log model evidence) is shown on the lower right; this is just a softmax function of the corresponding variational free energies (i.e., the exponential function of free energies normalized to a sum of one). This model comparison suggests that all three phenomenological parameters (change, frequency, and amplitude of global gamma responses) are useful under the neural field models considered, when explaining between subject variations in intrinsic connectivity.

### Bayesian Model Reduction

This Bayesian model comparison assesses the importance of different (combinations of) explanatory variables; however, this does not tell us which intrinsic connections are important for mediating individual differences. To address this, we then performed an exhaustive search over models examining all combinations of second level parameters. The second level parameters include the effects of the three explanatory variables on each of the (10) intrinsic connections (see Table 2). This means there are 30 parameters (ignoring the constant). In other words there is one second level parameter for each of the 10 parameters of the first level (neurobiological) model included in Table 2 and each of the three chosen phenotypes. Using Bayesian model reduction, we scored every combination of parameters to exclude redundant parameters and identified which intrinsic connections were responsible for mediating between subject effects (using **spm_dcm_peb_bmc.m**). In other words, we considered all possible hypotheses: each assuming a different combination of the connectivity parameters (see Table 2) could best explain intersubject variability in gamma responses.

Candidate models were obtained by removing one or more connections (arrows in Fig. 1) to produce restricted (reduced) forms of the full model that differ only in their priors. Bayesian model reduction then allowed us to score the evidence (and conditional densities) of each model, without having to explicitly invert the reduced models: see [Friston and Penny, 2011] for details.

**Figure 5:**
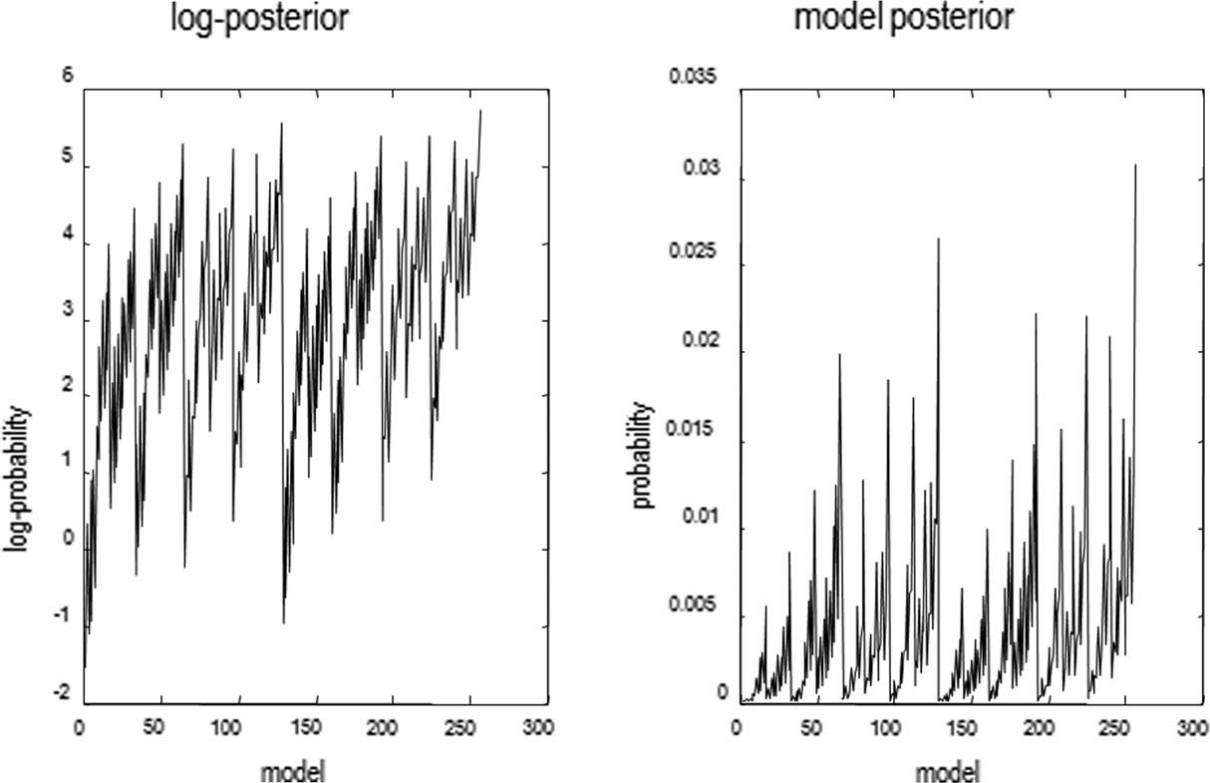
Log‐evidence over 256 most likely models (left) and associated posterior probabilities (right) following a greedy search over the parameter space characterizing the intrinsic connectivity of the microcircuitry in Figure 1.This considers all possible combinations of second level parameters and connections that might mediate between‐subject effects. Model likelihoods are effectively soft max functions of the corresponding variational free energies.

Figure 5 shows the log evidence over the most likely 256 models (following a greedy search) and the associated posterior probabilities (left and right panels respectively). In each of these (nested) models, one or more second level parameters [cf., Eq. (4)] have been removed by setting the prior covariance to zero. The log‐evidence of the resulting model is then computed using Bayesian model reduction [Friston et al., 2016]. This procedure considers all possible combinations of second level parameters and connections that might mediate between‐subject effects. Model likelihoods are effectively soft‐max functions of the corresponding variational free energies.

**Figure 6:**
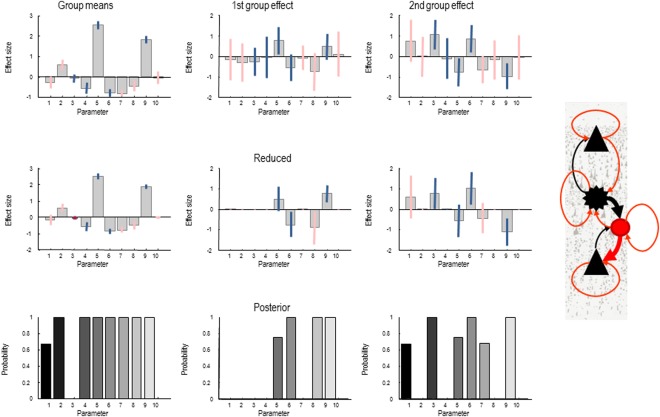
Posterior estimates before (upper panels) and after (lower panels) Bayesian model reduction. Second level effects comprised the group mean (left column) gamma change with size (first group effect – middle column) and gamma peak frequency (second group effect [right column]). Posterior means are in gray and 90% confidence intervals are depicted as colored bars. Note that the Bayesian model reduction has identified intrinsic connections that selectively involve II. Model posteriors for models with and without each second level parameter are shown in the lower row and indicate the probability that these effects are necessary to account for intersubject variability. See Table II for a list of the 10 parameters in this Figure and the CMC parameters of Figure 1. For clarity, we have highlighted connectivity parameters that involve II with a blue confidence bar. [Color figure can be viewed at http://wileyonlinelibrary.com]

The associated posterior estimates of the second level models are shown in Figure 6, before and after model reduction. The top row shows the parameter estimates before Bayesian model reduction in terms of their posterior means (gray bars) and 90% Bayesian confidence intervals (pink lines). These are the Bayesian equivalent of standard errors in classical inference. The second row shows the equivalent results after Bayesian model reduction; following which, some parameters have been removed because they are not necessary to explain the data. Positive and negative parameter estimates indicate the direction of the influence of the phenotype on subject‐specific connectivity. In this figure, second level effects corresponded to subject‐specific changes in gamma response with size and gamma peak frequency. The key thing to observe in Figure 6 is that many parameters have been eliminated during model comparison (or reduction). Here, the parameters have been separated into the group mean (first column) and the first two group effects (change and frequency), in the second and third columns respectively. The lower row reports the posterior probability of models with and without each (second level) parameter.

The remarkable thing about these results is that (with one exception), all the connections—that have been identified as mediating between subject effects—involve II. For example, intrinsic connections three, six and nine correspond to intrinsic connections from II to SS populations, deep pyramidal cells to II, and II to deep pyramidal cells respectively. Interestingly, the intrinsic connectivity from deep pyramidal cells to II (parameter 6 in Fig. 6) was previously implicated using a DCM of MEG data in a related context. We will focus on this connection in our final analysis.

In summary, we considered alternative hypotheses (models) where all possible combinations of these parameters might explain group differences and evaluated the evidence for these hypotheses using data from multiple subjects. Our results suggest that group variability emerges from differences in intrinsic connections that reflect individual differences in the excitation to inhibition balance.

### Cross‐Validation Predictive Validity

In the above analysis, we used empirical Bayes to equip the (first level) DCM parameters with optimal shrinkage priors to explain group effects in gamma response variability. In brief, we found that individual differences are mediated primarily by connections to and from the II. In the last analysis, we consider an alternative use of these (shrinkage) priors; namely, we treat empirical priors obtained by inverting a group of (training) subjects as full priors when inverting a new (test) subject that belongs to the same group. In other words, we evaluate the posterior belief about a model parameter in an unknown (test) subject (in particular the connection between II and deep pyramidal cells) and parameter estimates from the remaining subjects. In other words, we used all but one subject to estimate the model parameters (changes in effective connectivity) that best explained intersubject variations in spectral response to stimulus sizes. These estimates allowed us to use the parameter estimate from the “left out” subject to predict their spectral responses (i.e., spectral phenotype); based on, and only on, effective connectivity changes. By comparing the predicted and actual spectral responses for every “left out” subject, we can therefore establish an unbiased (out‐of‐sample') correlation between the effective connectivity and spectral phenotype.

In contrast to the analyses above, this approach reverses the roles of explanatory variables and model parameters in the design matrix—and can be used to establish the predictive validity of DCMs. This approach also allows us to quantify the extent to which connectivity from deep pyramidal cells to II (i.e., the sensitivity of II to interlaminar projections from deep pyramidal cells) explains intersubject differences in gamma responses. We used a leave one out scheme to provide a posterior predictive density over a range of gamma response changes. This provides an out‐of‐sample estimate of the intersubject variance that can be explained by just knowing a single (DCM estimate of) intrinsic connection strength. This analysis also serves as a cross validation because the posterior predictive density is based on independent data (in the leave one out scheme).

**Figure 7:**
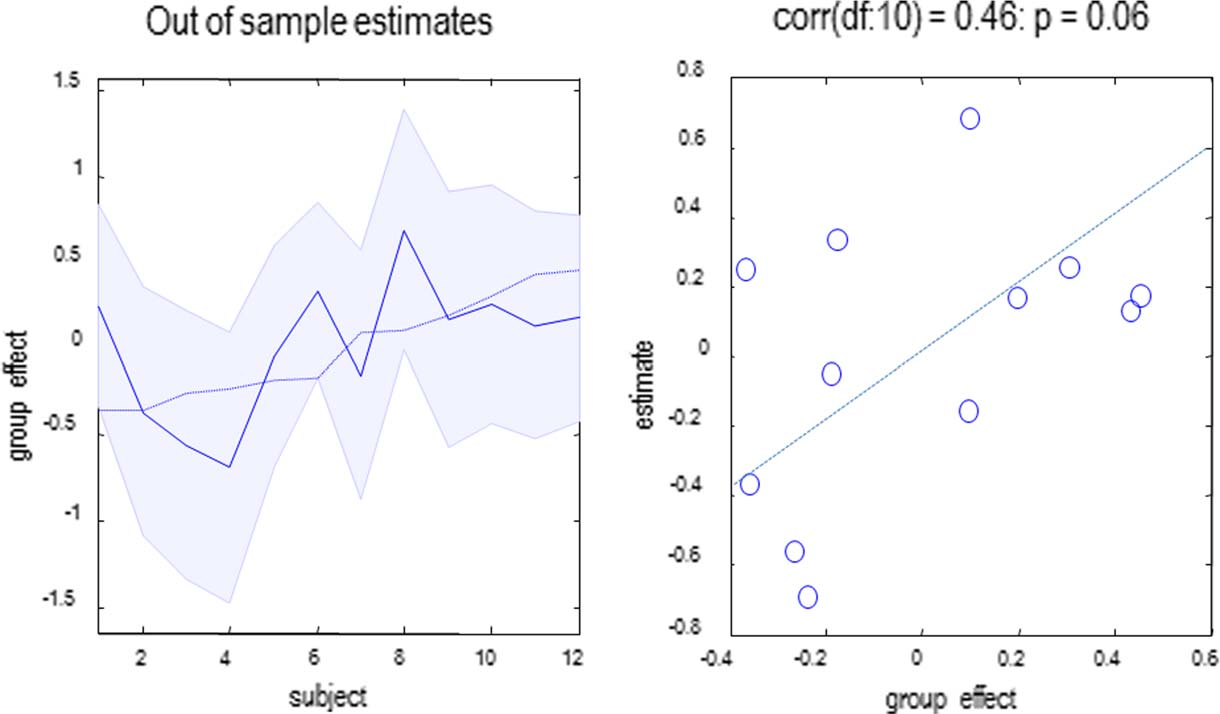
Leave‐one‐out analysis illustrating Bayesian cross‐validation. The left panel shows the predicted (posterior) gamma response change for each subject, based on parameter estimates obtained from the remaining subjects. The subjects have been ordered to show an increasing gamma response change with stimulus size. The right panel shows the expected and observed gamma response change estimates and their correlation (0.46, *P* = 0.06). Note that this correlation was obtained using independent (non‐invasive) MEG beam‐formed data. [Color figure can be viewed at http://wileyonlinelibrary.com]

Figure 7 shows the results of this analysis (using **spm_dcm_loo.m**). The left panel shows the true and (out‐of‐sample) predicted gamma response change (after mean correction and Euclidean normalization). The shaded area represents the 90% Bayesian confidence intervals. These estimates are predictive in the sense that they are based on data that did not include the subject being predicted. In other words, these represent out‐of‐sample estimates that reflect what would happen if we were just given the estimate of intrinsic connectivity from a new subject. The correlation between the expected and the observed response change was 0.46, which means one could explain over 20% of intersubject variability in stimulus size selective gamma responses, given the posterior estimate of intrinsic connectivity from deep pyramidal cells to II. The significance of this correlation was trend significant (*P* = 0.06) and can be regarded as a cross‐validation accuracy verification of the Bayesian model reduction. Note that although the significance of this correlation is only trend significant (*P* < 0.06) it reflects a conservative out‐of‐sample correlation. In other words, this is the correlation we would expect with a new cohort of independent subjects based on the parameters estimates from the current subjects.

In addition to predicting phenotypic traits using cross validation, one can also use the optimized model to generate physiological responses (at the single subject or group level). This can be useful for examining how various connectivity (or condition‐specific changes) contribute to observable spectral responses. Indeed, generating predicted responses from optimized DCM can also be used to ask which data features are important for informing estimates of specific model parameters.

## DISCUSSION

Biophysical modeling of brain activity has a long history [Coombes, 2010; Deco et al., 2008; Ermentrout, 1998] and has recently profited from technological advances that furnish neuroimaging data at an unprecedented spatiotemporal resolution [Guillory and Bujarski, 2014; Pinotsis and Friston, 2014c; Sporns, 2014]. Neuronal modeling is a very active area of research, with applications ranging from the characterization of neurobiological and cognitive processes, [Bojak and Liley, 2005; Jirsa, 2004a, 2004b; Phillips and Robinson, 2009; Rolls and Treves, 2011] to constructing artificial brains in silico and building brain‐machine interface and neuroprosthetic devices, for example, [Einevoll et al., 2013; Whalen et al., 2013].

Here, we used an important class of biophysical models called ‘neural fields’ in the DCM framework to characterize individual differences in induced responses in the gamma band during a visual perception experiment. In this analysis, neural fields constitute the first level of a hierarchical Bayesian model, acting like a filter that receives spatially uncorrelated (scale‐free) noise as temporal input and produces output spectra; notably in the gamma band. Modulations of these responses across individuals reveal differences in subject‐specific anatomy and physiology captured by the neural field parameters. In particular, we asked whether variability in stimulus size, tuning, and gamma responses can be explained by intersubject differences in intrinsic connections forming a PING network. We found strong evidence in non‐invasive (spectral) data for this hypothesis. Furthermore, Bayesian model comparison (at the between‐subject level) identified the connections accounting for intersubject variability and these were exclusively to and from II. This finding is supported by a number of theoretical and empirical lines of argument: we focus on three perspectives that explain why inhibitory connections are implicated in visually induced gamma responses:

First, from a theoretical perspective, one can appeal to a predictive coding explanation for individual differences in gamma band activity: in this setting, size effects may be mediated by changes in cortical excitability and, ultimately, the gain of pyramidal cell populations [Adesnik et al., 2012; Alitto and Usrey, 2008], which optimize the precision or gain of units encoding visual prediction errors: see [Pinotsis et al., 2014] for a discussion of the role of gain and precision in contrast effects. The excitability of principal cell populations is thought to change as a result of feedback connections from extrastriate areas that exert modulatory effects [Angelucci and Bressloff, 2006; Rockland and Pandya, 1979; Shao and Burkhalter, 1996] on responses to forward LGN input [Sadakane et al., 2006; Solomon et al., 2006].

Physiologically, the changes in the post‐synaptic gain of neuronal populations are most likely due to changes in local inhibition in a manner reminiscent of PING models [Traub et al., 1997; Tiesinga and Sejnowski, 2009]. In this context, the activity of inhibitory cells within the visual network depends on stimulus condition and different sizes lead to changes in the excitation to inhibition balance—and weak suppression of interneurons [Haider et al., 2010]. Changes in the amplitude of gamma oscillations may be due to interactions between principal cells and interneurons as the grating patch expands beyond the classical receptive field. These interactions are thought to be mediated by local intrinsic (horizontal) connectivity that might drive gamma oscillations [Cunningham et al., 2004; Whittington et al., 1995] and alter their form and coherence [Jia et al., 2013; Ray and Maunsell, 2010].

These gain control mechanisms may also mediate surround suppression—through changes in spatial integration properties. In the primary visual cortex, excitatory and inhibitory pools of neurons are proximally located and connected through elongated axonal collaterals. This implies that the boundary between the effective extent of excitation and inhibition—the relative densities of neurons of different types that are activated for various stimuli—is flexible. This can also reveal the functional specificity of gamma rhythms for different stimulus sizes and suggest an approximate distance over which local generators might show synchronization [Leopold et al., 2003].

Our findings are also in accord with recent computational work modeling the emergence of gamma power peaks in the visual cortex based on an inhibition‐stabilized network [Jadi and Sejnowski, 2014a; Tsodyks et al., 1997], that exhibits an Andronov–Hopf bifurcation: see [Pinotsis et al., 2012] for more details. In this article, we used neural fields to model the dispersion of axonal connections and describe the effect of surround suppression on gamma responses, see also [Pinotsis et al., 2013, 2014]. Finally, our source model comprises four populations whose connectivity is similar to recent theoretical and experimental work that focuses on the origins of visually induced gamma peak [Jadi and Sejnowski, 2014b; Ozeki et al., 2009].

The neurobiological mechanisms considered above highlight the central role of inhibitory connectivity in generating gamma response variability [Cardin et al., 2009] and regulating PING activity [Brunel and Wang, 2003; Gieselmann and Thiele, 2008; Ray and Maunsell, 2010]. It should be noted that these mechanisms are not mutually exclusive and might coexist or contribute to a different extent depending on individual differences and experimental conditions [Webb et al., 2005; Liu et al., 2011]. Our model predicts these relative contributions by estimating different effective connectivity weights that describe individual differences in cortical anatomy and synaptic efficacy. Our conclusion is that gamma responses reflect the balance of input to the local excitatory and inhibitory neurons and individual differences in a PING network with strong excitatory–inhibitory feedback, see also [Wallace et al., 2011].

This article serves to introduce hierarchical modeling for DCM studies of electromagnetic responses. Our example focused on the underlying connectivity generating responses to visual stimuli (of the same size)—trying to explain intersubject variability in (intrinsic) connectivity, in terms of subject specific differences in gamma responses to stimuli (of different sizes). More refined analyses of intersubject variability could consider the effect of stimulus size on intrinsic connectivity. In other words, one can apply the same procedures not just to connectivity but to context‐sensitive changes in connectivity elicited at the within subject level. In the present example, this would involve fitting responses to all three stimulus sizes and parameterizing the effect of stimulus size on intrinsic connectivity: see Pinotsis et al [2014] for an example of this sort of parameterization at the within subject level.

The analysis showcased in this work uses a general empirical Bayesian framework for model comparison at the group level: see [Friston et al., 2016]. These procedures use Bayesian model reduction to provide a generalization of the summary statistic approach to nonlinear (e.g., dynamic causal) models and to evaluate the evidence of large sets of nested models. Finally, the same statistical technology was used for Bayesian cross‐validation—and to obtain predictive estimates for gamma‐response phenotypes using a leave‐one‐out procedure. This is the first application of empirical Bayes for DCM using real (electrophysiological) data; similar analyses may help establish the usefulness of this approach—and the predictive validity of DCM—in clinical and pharmacological settings.

## Footnotes

1 Throughout this article, we assume rotational symmetry and use the usual convention for the Fourier transform of a function v(x,t)

$V(k,\omega )=\int_{-\infty }^{\infty }dx\int_{0}^{\infty }dtv(x,t)e^{-ikx+\omega t},\;\;\;\;\;\omega \in \theta ,k\in \mathbb{R}$ where k and ω are the spatial and temporal frequencies, respectively, and the spatial variable *x* is one dimensional. Also, the sum in Eq. (2.2), follows from an assumption about periodic boundary conditions under which cortical activity can be viewed as a superposition of standing waves of various spatial frequencies, for more details see [Pinotsis et al., 2012].

2 Software note: References to .m files here correspond to Matlab files included in the current version of SPM12 (http://www.fil.ion.ucl.ac.uk/spm/).

## Appendix

In Eq. (2), $T(k,\omega ,\theta^{\left(1\right)})=T(k,\omega )=[T_{1}(k,\omega ),\ldots ,T_{4}(k,\omega )]$ where we drop the explicit mention of $\theta^{\left(1\right)}$ in the argument. The transfer functions $T_{a}(k,\omega )$ mediating the contribution of individual populations to measured responses are given by the following expressions:

(A.1) $$T_{a}(k,\omega )=\kappa_{1}W^{-1}(k,\omega )Z_{a}(k,\omega )$$

The transfer functions express the relative contribution of each population to the predictions at the sensor level and depend on the particular form of the connections among source populations, where W(k,ω) and $Z_{a}(k,\omega )$ are given by

(A.2) $$\begin{array}{c} W(k,\omega )=-R_{14}(k,\omega )(-R_{23}(k,\omega )+P_{3}(k,\omega )P_{2}(k,\omega ))\\ +P_{4}(k,\omega )[-R_{23}(k,\omega )P_{1}(k,\omega )+P_{3}(k,\omega )(-R_{12}(k,\omega )+P_{2}(k,\omega )P_{1}(k,\omega ))]\\ Z_{1}(k,\omega )=-P_{4}(k,\omega )(-R_{23}(k,\omega )+P_{3}(k,\omega )P_{2}(k,\omega ))\\ Z_{2}(k,\omega )=D_{21}(k,\omega )\gamma \kappa_{2}P_{4}(k,\omega )P_{3}(k,\omega )\\ Z_{3}(k,\omega )=-D_{21}(k,\omega )D_{32}(k,\omega )\gamma^{2}\kappa_{2}\kappa_{3}P_{4}(k,\omega )\\ Z_{4}(k,\omega )=D_{41}(k,\omega )\gamma \kappa_{4}(-R_{23}(k,\omega )+P_{3}(k,\omega )P_{2}(k,\omega )) \end{array}$$

and the functions $P_{a}(k,\omega )$ and $R_{ab}(k,\omega )$ are given in terms of the Fourier transforms $D_{ab}^{}(k,\omega )$ as follows:

(A.3) $$\begin{array}{c} P_{a}(k,\omega )=2i\kappa_{a}\omega +\omega^{2}-\kappa_{a}{}^{2}+\gamma D_{aa}^{}(k,\omega )\kappa_{a}\\ R_{ab}(k,\omega )=\gamma^{2}\kappa_{a}\kappa_{b}D_{ab}^{}(k,\omega )D_{ba}^{}(k,\omega ) \end{array}$$

where $\kappa_{a}$ are the post synaptic time constants and γ is neuronal gain.
